# Mother to child transmission of HIV in Plateau State, Nigeria: Case-based surveillance of the HIV infected HIV-exposed infants

**DOI:** 10.1371/journal.pone.0350012

**Published:** 2026-06-23

**Authors:** Emmanuel O. Osayi, Sarah C. Blake, Kelechi Ngwoke, Catherine Pius, Tolulope Afolaranmi, Atiene S. Sagay

**Affiliations:** 1 APIN Public Health Initiatives, Jos, Plateau, Nigeria; 2 Rollins School of Public Health United States of America, Emory University, Atlanta, Georgia, United States of America; 3 APIN Public Health Initiatives, FCT, Abuja, Nigeria; 4 Jos University Teaching Hospital, Jos, Plateau, Nigeria; SKYDA Health Nigeria, NIGERIA

## Abstract

**Introduction:**

HIV infection among children is predominantly due to perinatal transmission. The timing of HIV-related symptoms in perinatally infected children reveals how quickly the disease progresses and helps predict prognosis. This study used case-based surveillance to examine treatment outcomes and disease progression among HIV-infected, HIV-exposed infants in Plateau State, Nigeria.

**Method:**

This is a retrospective cohort study using a case-based surveillance approach. The data of HIV-exposed infants enrolled between 1^st^ October 2018 and 30^th^ September 2022, who had confirmed positive DNA PCR results and commenced antiretroviral therapy (ART), were sampled via a cluster sampling method. Participants were followed up for 6 months to 5 years post-ART initiation. Disease progression, treatment mortality, and loss outcomes were assessed with respect to age at follow-up.

**Results:**

A total of 57 infants were included in the study, drawn from 15 health facilities across the three senatorial districts of Plateau State, Nigeria. The female-to-male ratio was approximately 1:1 (29:28), with a mean age of 0.7 years (8.9 months) ± 0.7 years at ART initiation. By the end of the follow-up period, 70% (40/57) of the infants had favorable treatment outcomes, with 87.5% achieving viral suppression. The remaining 29.8% (17/57) experienced treatment mortality or losses (TX_ML): 47.1% (8/17) died, 29.4% (5/17) transferred out, 17.6% (3/17) discontinued treatment, and 5.9% (1/17) interrupted treatment. Among those with mortality outcomes, 75% died before the age of two years, while 25% survived beyond two years, with some reaching up to their fourth birthday. Mortality was unevenly distributed across local government areas (LGAs), with the highest rates recorded in Mangu LGA (37.5%), followed by Shendam LGA (25%), and the remaining 37.5% shared equally among Pankshin, Langtang North, and Jos North.

**Conclusion:**

This study revealed disparities in TX_ML outcomes across senatorial districts, with higher treatment mortality in the Southern and Central districts compared to the Northern district. These findings highlight the need to design targeted, context-specific interventions to improve survival and treatment outcomes among perinatally HIV-infected children and further research to identify the contributing factors.

## Introduction

Perinatal HIV infection, also called mother-to-child transmission of HIV, occurs during the perinatal period of a pregnancy, which is from 22 weeks of gestational age up to seven days after birth [1]. The infection occurs during pregnancy, labour and delivery, breastfeeding, and postpartum, with an estimated 2.6 million children 0–9 years reported living with HIV, while the new infection rate among the same age group was approximately 48 percent [2]. Similarly, the Nigeria HIV/AIDS Impact and Indicator Survey (NAIIS) reported the prevalence of HIV among children ages 0–14 as 0.2 percent, with a significant number of the infections occurring during the perinatal period.

Furthermore, the rapidity of the HIV disease progression and prognosis among perinatally infected children could be categorized based on the severity of HIV-related symptoms. About 25–30 percent of the perinatally infected children are known as rapid progressors because they rapidly progress to HIV and AIDS, and the infection is mostly in-utero [2,3]. Another 50–60 percent, known as mid progressors, grow to one year old but rapidly deteriorate and die, while an estimated proportion of perinatally infected children (8–25 percent), known as long-term survivors, go beyond eight years of age [3]. High mortality rates have been recorded among rapid progressors, followed by mid progressors [1–3], with most of the deaths due to intercurrent infections and malnutrition, which, with appropriate surveillance and timely intervention, would propel them to long-term survivors and beyond.

To better understand the HIV disease progression, the treatment mortality, and loss outcomes among infants born to HIV positive women with positive HIV status in Plateau State, Nigeria, a case-based surveillance approach was adopted for the study [4].

## Study materials and methods

### Study location

The study was conducted in Jos, Plateau State, Nigeria, which covers 26,899 square kilometers and is bounded by Bauchi (northeast), Kaduna (northwest), Nasarawa (southwest), and Taraba (southeast). Administratively, the State is divided into three senatorial districts—Northern, Central, and Southern. According to the 2025 Annual Operational Plan, 1,233 health facilities serve the population, with about 3.2% (n = 40) providing Prevention of Mother-to-Child Transmission of HIV (PMTCT) services to confirmed HIV-positive pregnant women through donor support. Plateau State has a HIV prevalence of 1.5%, slightly higher than the national prevalence of 1.4%, with notable gender differences: among adults aged 35–39 years, women have more than double the prevalence (3.1%) compared to men (1.4%) [5,6], a disparity attributed to population distribution by sex at birth and women’s relatively higher health-seeking behavior.

### The study method and sampling technique

This study employed a retrospective cohort design and used a cluster sampling technique to include infants born to HIV-positive women (HIV-exposed infants) enrolled into care between 1st October 2018 and 30^th^ September 2022, with confirmed HIV positive DNA PCR results. This cluster was across 15 comprehensive ART facilities in Plateau State, Nigeria, and they provide quality HIV care and treatment to more than 50 percent of PLHIV in Plateau State, Nigeria.

### The study's inclusion and exclusion criteria

**Fig 1:**
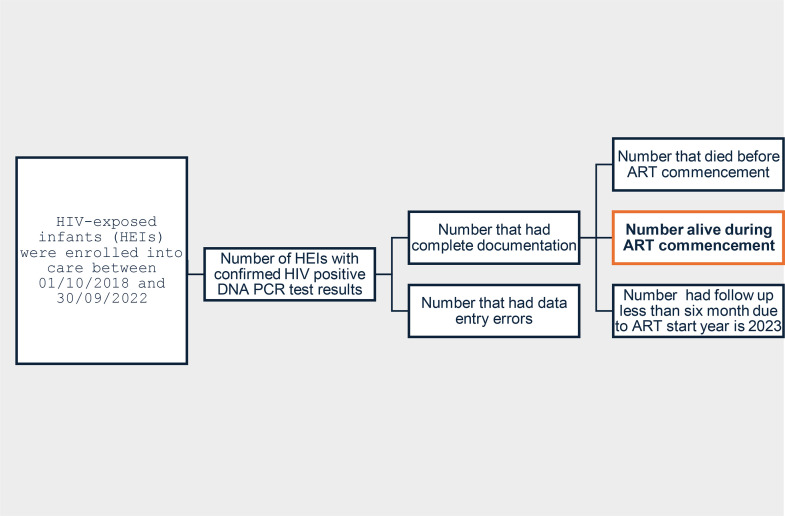
Schematic diagram of the study eligibility criteria.

HIV infection was confirmed with a positive DNA PCR test, which was validated by a second DNA PCR sample collected during enrollment. Infants were initiated on age-appropriate antiretroviral therapy (ART) following confirmation. Only HIV-positive infants with complete documentation and active follow-up at the time of the study were included. Infants were excluded if they had incomplete data (e.g., date of birth, date of confirmed HIV diagnosis, or date of ART initiation), died before starting ART, had less than six months of follow-up from ART initiation, or met a combination of these criteria. Participants were followed up for a minimum of six months and up to five years post-ART initiation. See details in Fig 1.

### Ethical consideration and consent

Ethical approval for the use of secondary program data was obtained from the Health Research Ethics Committee (Approval Number: NHREC/09/23/2010b) and covered all participating health facilities. Written informed consent had been obtained from the mothers/guardians of the infants at the time of enrolment into care, which included permission for the use of de-identified secondary data for research purposes. To ensure confidentiality, all data were anonymized before analysis and were accessible only to authorized study personnel.

### Data collection and analysis

A proforma was used to extract the study data from the health facilities’ child follow-up registers and clients’ care cards on 10^th^ September 2025. The patient-level variables collected were the infants’ unique identification numbers, date identified positive, age at ART start, sex, mode of HIV detection, local government area of antiretroviral therapy (ART) initiation, viral load result, and treatment outcome.

The outcome variables of interest were the treatment mortality and losses (TX_ML), which expressed client interruption in treatment (IIT), death, stop, and transfer out, and viral load results [7].

TX_ML_ Interruption in treatment was defined as the patient being unable to locate and pending return to the facility [7]. TX_ML_ Transfer out was defined as successful when the client was confirmed to have successfully transferred to another facility, TX_ML_ stop when the client contacted, confirm stopping/discontinuing the antiretroviral therapy within the period, followed up, and TX_ML_ death variable was defined based on verbal autopsy from the clients’ caregivers [7]. To clearly define the distribution of the TX_ML outcomes by participants’ duration on ART, duration on ART was categorized into < 2 years and ≥ 2 years. The participants’ status at the end of the study was defined as active (HIV positive on ART with no TX_ML outcomes) and inactive (HIV positive on ART with any of the TX_ML outcomes).

The variable viral load result was dichotomized to virally suppressed, less than 1000 copies per ml, and virally unsuppressed, ≥ 1000 copies per ml. The virally suppressed were further disaggregated to undetectable, less than 50 copies per ml. The most recent viral load test result was used in monitoring the disease progression by the end of the follow-up period.

A case-by-case narrative was used to analyze the de-identified data, including frequencies, proportions, mean, median, interquartile range, and corresponding standard deviation calculated using an Excel spreadsheet. The authors had access to information that could identify the individual participants during or after data collection.

## Results

### Study participants

A total of 5761 HIV-exposed infants were enrolled into care between October 2018 and September 2022 in 15 health facilities across the three senatorial districts in Plateau State, Nigeria. Out of which, 86 infants had confirmed HIV positive DNA PCR test results, giving an MTCT rate of 1.5%.

Of the 86 infants with confirmed HIV positive DNA PCR test results, 77.9% (67/86) had complete documentation, while 22.1% (19/86) had data entry errors.

**Fig 2:**
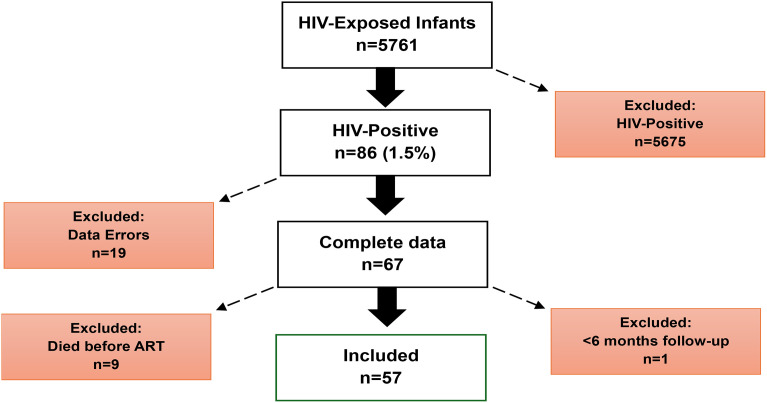
Flow Diagram of the Study Population Inclusion.

Among the 67 HEIs with complete documentation, 13.4% (9/67) died before ART commencement, 1.5% (1/67) had follow-up of less than 6 months because the ART start year was 2023, and the remaining 85.1% (57/67) met the study eligibility criteria and were recruited. See details in Fig 2.

The study included 57 infants, with a nearly equal female-to-male ratio (29:28). See further details in Table 1 below. The mean age at ART initiation was 8.9 months. By the end of the follow-up period, approximately 70% of the infants remained active in care, while 30% were classified as inactive.

**Table 1:** Socio-demographics of the eligible study HEIs (n = 57).

Socio-demographic characteristics	Total
**Sex**	
Male	**28 (49.1)**
Female	**29 (50.9)**
**LGAs distribution**	
Jos North	**19 (33.4)**
Jos South	**4 (7.0)**
Barkin Ladi	**2 (3.6)**
Bokkos	**5 (8.7)**
Kanam	**2 (3.5)**
Mangu	**10 (17.5)**
Pankshin	**3 (5.3)**
Langtang North	**6 (10.5)**
Shendam	**6 (10.5)**
**Senatorial Districts distribution**	
Northern	**25 (43.9)**
Central	**20 (35.1)**
Southern	**12 (21.0)**
**Status at the end of the review period**	
Active	**40 (70.2)**
Inactive	**17 (29.8)**

Inactive participants were classified according to their TX_ML outcomes. The most frequent outcome was death (47.1%), followed by transfer out (29.4%), discontinued/stopped ART (17.6%), and treatment interruption (5.9%), which was the least common outcome.

Using the ART initiation date and the last drug pick-up date as reference points, the study assessed the duration participants remained on ART before their TX_ML outcome occurred. Approximately 65% of inactive participants had been on ART for less than two years, with ‘Died’ accounting for about 45% of the total outcomes within this group. The remaining 35% had been on ART for two years or longer before their TX_ML outcome. Further details are presented in Table 2.

**Table 2:** Distribution of the TX_ML outcomes by duration on ART (n = 17).

Duration on ART	Died	Transferred Out	Interruption in Treatment	Stopped / Discontinued Treatment	Total (%)
< 2 years	5	3	0	3	11 (64.7)
2 years and above	3	2	1	0	6 (35.3)
Total (%)	8	5	1	3	17 (100)

Similarly, using date of birth and last drug pick-up date as reference points, the study examined the age distribution of inactive participants at the time of their last drug pick-up before the TX_ML outcome. Approximately 71% of participants were aged two years and above, with ‘Died’ representing half of the total outcomes, followed by transfer out (33.3%). In contrast, the remaining 29.4% of participants were younger than two years of age at the time their TX_ML outcome was recorded. Additional details are provided in Table 3.

**Table 3:** Distribution of the TX_ML outcomes by Age (n = 17).

Age	Died	Transferred Out	Interruption in Treatment	Stopped / Discontinued Treatment	Total (%)
< 2 years	2	1	0	2	5 (29.4)
2 years and above	6	4	1	1	12 (70.6)
Total (%)	8	5	1	3	17 (100)

Furthermore, the study reported an equal sex distribution of the TX_ML died outcome, with the Central Senatorial district having half of the burden, followed by the Southern Senatorial district (37.5%), and the least, the Northern Senatorial district (12.5%). The burden of TX_ML died outcome in the central senatorial district was reported to be highest in Mangu Local Government Area (LGA), while that in the Southern Senatorial district was reported in Shendam LGA. See Table 4 below.

**Table 4:** Sociodemographic distribution of the mortality per year of study (n = 8).

Sociodemographic characteristics	Total (%)
**Sex**	
Male	**4(50.0)**
Female	**4(50.0)**
**Senatorial District**	
Northern	**1(12.5)**
Central	**4(50.0)**
Southern	**3(37.5)**
**Local Government Areas**	
Mangu	**3 (37.5)**
Jos North	**1 (12.5)**
Shendam	**2 (25.0)**
Pankshin	**1 (12.5)**
Langtang North	**1 (12.5)**

For the active participants, valid viral load results were used to monitor their disease progression. All the latter had valid viral load results as at the end of the follow-up period, with approximately 13% virally unsuppressed (had viral load results greater than 1000 copies per ml), while 87.5% were virally suppressed (had viral load results less than 1000 copies per ml). Among the virally suppressed, 86 percent had an undetectable viral load result. The study showed that most of the participants virally unsuppressed were female sex, in the central senatorial district, while those virally suppressed were equally distributed by sex and more in the northern senatorial district, specifically Jos North LGA. See Table 5 below.

**Table 5:** The sociodemographic of the disease progression among active participants (n = 40).

Variables	≥ 1000 copies/ ml (%)	< 1000 copies/ml (%)
Viral load result	5 (12.5)	35 (87.5)
Sex		
Male	2 (5.0)	17 (42.5)
Female	3 (7.5)	18 (45.0)
Senatorial District		
Northern	2 (5.0)	18 (45.0)
Central	3 (7.5)	10 (25.0)
Southern	0 (0.0)	7 (17.5)
Local Government Area		
Jos North	2 (5.0)	14 (35.0)
Jos South	0 (0.0)	1 (2.5)
Bladi	0 (0.0)	3 (7.5)
Mangu	2 (5.0)	4 (10.0)
Bokkos	0 (0.0)	4 (10.0)
Kanam	1 (2.5)	1 (2.5)
Pankshin	0 (0.0)	1 (2.5)
Shendam	0 (0.0)	3 (7.5)
Langtang North	0 (0.0)	4 (10.0)

## Discussion

Prevention of Mother-to-Child Transmission (PMTCT) of HIV remains one of the most significant global public health interventions to reduce vertical HIV transmission and to achieve the UNAIDS goal of reducing mother-to-child transmission (MTCT) to less than 5% [8]. The findings of this study align with that global vision. The MTCT rate reported was 1.5% among HIV-exposed infants (HEIs) enrolled into care during the review period. This figure is considerably lower than the reported national pooled MTCT rate of 2.7% and demonstrates that the PMTCT program in Plateau State has matured into a highly effective and contextually responsive model. The success highlights the program’s strategic mitigation of risk factors associated with MTCT, particularly through strengthened antenatal care, ART provision, and community-level interventions aimed at eliminating pediatric HIV.

Despite these positive strides, the study underscores persistent challenges that continue to undermine the long-term success of HIV care and treatment programs, particularly the issue of treatment losses. Globally, attrition from antiretroviral therapy (ART) is a widely recognized barrier to sustained epidemic control, and developing countries like Nigeria are disproportionately affected. By the end of the study’s follow-up period, approximately 50% of the inactive participants experienced one or more forms of treatment losses, whether transferred out, discontinued treatment, or experienced treatment interruption. Independent factors significantly associated with these losses included biological or caregiver responsibility for the children, as well as adherence challenges. Importantly, the fact that nearly two-thirds of these losses occurred within the first two years of ART initiation highlights a vulnerable window in early treatment where patients require intensified follow-up, adherence support, and psychosocial interventions [9,10].

The study also sheds light on the grave issue of disease progression and mortality among HIV-infected children. Globally, disease progression in people living with HIV, particularly children, has been linked to advanced HIV disease and early mortality [11, 12]. In this cohort, approximately half of the inactive participants died during the follow-up period, with a significant proportion succumbing before their second birthday. This early mortality underscores the rapid progression of HIV disease in infants and the urgent need for timely diagnosis, early ART initiation, and robust clinical support systems. On the other hand, those who lived beyond two years, up to their fourth birthday, reflected a somewhat slower progression of disease, though still vulnerable. The unequal geographic distribution of mortality, with the central senatorial district—particularly Mangu LGA—bearing the highest burden, suggests potential disparities in healthcare access, resource allocation, and program delivery across Plateau State. This pattern raises critical questions about equity in health service distribution and the need for targeted resource deployment to underserved areas [11,13–15].

Monitoring viral load remains central to evaluating ART program performance and ensuring treatment success [3]. In this study, approximately 70% of the participants remained active in care at the end of the follow-up period, with most achieving viral suppression. Notably, 86% of these virally suppressed participants recorded undetectable viral loads (<50 copies/ml), a benchmark of ART effectiveness. Geographic patterns again emerged, with a high proportion of undetectable viral load results concentrated in the northern senatorial district, particularly Jos North. The cosmopolitan nature of this district, characterized by diverse cultural dynamics and potentially lower stigma, may contribute to higher treatment uptake and retention [16]. This observation illustrates the influence of social determinants, such as stigma and community norms, on program outcomes and emphasizes the importance of addressing structural and societal barriers to HIV care.

### Study limitations

While this study successfully demonstrated the importance of treatment interventions on disease progression and health outcomes among HIV-exposed infants with HIV-positive status, several limitations were identified. The study employed a cluster sampling method, which was practical for reaching participants across multiple facilities. However, the sample size was relatively small, and extending the study period could enhance the robustness of the findings. In addition, documentation errors identified in the source records affected the final sample size, although data quality has improved substantially in recent times.

## Conclusion

This study presents a dual narrative of progress and persisting gaps. The remarkably low MTCT rate demonstrates the effectiveness of the PMTCT strategies implemented in Plateau State, placing the region ahead of national averages and closer to the global elimination target. However, it reveals the challenges of ART attrition, early mortality, and geographical disparities, which remind us that success in HIV programming must be multidimensional, addressing not only biomedical interventions but also health system, societal, and caregiver-related factors.

## Supporting information

S1 Checklist PLOSOne_Human_Subjects_Research_Checklist. (DOCX)
